# Tetramer based approach for efficient identification and isolation of neo-antigen specific CD8 T cells from peripheral blood (PBL) of patients with metastatic cancers

**DOI:** 10.1186/2051-1426-3-S2-P47

**Published:** 2015-11-04

**Authors:** Mini Bharathan, Katarina Trebska-McGowan, Pasetto Anna, Drew C Deniger, Ken-ichi Hanada, Jared J Gartner, James C Yang, Steven A Rosenberg, Paul F Robbins

**Affiliations:** 1 NIH/NCI, Bethesda, MD, USA; 2 NIH/NCI Surgery Branch, Bethesda, MD, USA; 3 National Institutes of Health, Bethesda, MD, USA

## Background

Adoptive cell therapy with T cells bearing mutation specific T cell receptors (TCR) can be an effective method for treating metastatic cancers. The objective of this study was to identify mutation reactive T cells in the circulation of patients with different types of metastatic cancer.

## Methods

The strategy utilized whole exome sequencing data to identify somatic non-synonymous mutations and then in-silico algorithms to predict minimal epitopes encoding mutated amino acids for each patient specific HLA-allele. CD8-enriched PBL from each patient were stained with tetramers generated in house by a UV-exchangeable technique as previously described for A*02:01, A*03:01, A*11:01, B*07:02, and a commercial tetramer was acquired for B*57:01. Based on the initial staining frequency (+tetramer^+^ T cells recognizing 7 unique neo-antigens from the PBL of 4 patients (ranging from 1 to 4 per patient). We enriched the frequencies of CD8^+^tetramer^+^ cells from 0.5 to >85%, 0.3 to >65% and 0.01 to 3% from the PBL of patients with colorectal (3971-A*02:01), lung (4014-B*57:01), and ovarian (4067-B*07:02) cancers respectively, using individual tetramers. Populations reactive with three HLA-A*11:0-restricted and one HLA-A*03:01-restricted neoantigens were also isolated from the PBL of lung cancer patients 4014 and 4037, respectively, using a pooled tetramer approach.

## Results

Overall, the isolated T cells recognized mutated epitopes when co-cultured with autologous CD14^+^ monocytes pulsed with mutated peptides in the context of appropriate MHC-I alleles including HLA-A*02:01, HLA-A*03:01, HLA-A*11:01, HLA-B*07:02, and HLA-B*57:01, with reactivity detected using IFN-γ ELISA. Using single cell PCR, we could clone the TCRs reactive with an HLA-*02:01-presented colon cancer neoantigen and an HLA-B*57:01-presented lung cancer neoantigen. Evaluation of PBL retrovirally-transduced with these TCRs demonstrated that they bound to tetramers and secreted IFN-γ when co-cultured with CD14^+^ monocytes pulsed with appropriate mutated peptides.

## Conclusions

To conclude, tetramers offer a sensitive, fast and reliable methodology to isolate mutation specific tumor reactive T cells from PBL of cancer patients. Furthermore, this method facilitates the identification, and cloning of mutation reactive TCR with which to construct receptor-engineered T cells for adoptive T cell therapy.

